# Evolutionary consequences of polyploidy in prokaryotes and the origin of mitosis and meiosis

**DOI:** 10.1186/s13062-016-0131-8

**Published:** 2016-06-08

**Authors:** Alexander V. Markov, Ilya S. Kaznacheev

**Affiliations:** Biological Faculty, Department of Biological Evolution, M.V. Lomonosov Moscow State University, Leninskie Gory, 1, Bldg. 12, Moscow, 119991 Russia

**Keywords:** Evolution of sex, Origin of eukaryotes, Mitosis, Meiosis, Lateral gene transfer, Recombination, Polyploidy

## Abstract

**Background:**

The origin of eukaryote-specific traits such as mitosis and sexual reproduction remains disputable. There is growing evidence that both mitosis and eukaryotic sex (i.e., the alternation of syngamy and meiosis) may have already existed in the basal eukaryotes. The mating system of the halophilic archaeon *Haloferax volcanii* probably represents an intermediate stage between typical prokaryotic and eukaryotic sex. *H. volcanii* is highly polyploid, as well as many other Archaea. Here, we use computer simulation to explore genetic and evolutionary outcomes of polyploidy in amitotic prokaryotes and its possible role in the origin of mitosis, meiosis and eukaryotic sex.

**Results:**

Modeling suggests that polyploidy can confer strong short-term evolutionary advantage to amitotic prokaryotes. However, it also promotes the accumulation of recessive deleterious mutations and the risk of extinction in the long term, especially in highly mutagenic environment. There are several possible strategies that amitotic polyploids can use in order to reduce the genetic costs of polyploidy while retaining its benefits. Interestingly, most of these strategies resemble different components or aspects of eukaryotic sex. They include asexual ploidy cycles, equalization of genome copies by gene conversion, high-frequency lateral gene transfer between relatives, chromosome exchange coupled with homologous recombination, and the evolution of more accurate chromosome distribution during cell division (mitosis). Acquisition of mitosis by an amitotic polyploid results in chromosome diversification and specialization. Ultimately, it transforms a polyploid cell into a functionally monoploid one with multiple unique, highly redundant chromosomes. Specialization of chromosomes makes the previously evolved modes of promiscuous chromosome shuffling deleterious. This can result in selective pressure to develop accurate mechanisms of homolog pairing, and, ultimately, meiosis.

**Conclusion:**

Emergence of mitosis and the first evolutionary steps towards eukaryotic sex could have taken place in the ancestral polyploid, amitotic proto-eukaryotes, as they were struggling to survive in the highly mutagenic environment of the Early Proterozoic shallow water microbial communities, through the succession of the following stages: (1) acquisition of high-frequency between-individual genetic exchange coupled with homologous recombination; (2) acquisition of mitosis, followed by rapid chromosome diversification and specialization; (3) evolution of homolog synapsis and meiosis. Additional evidence compatible with this scenario includes mass acquisition of new families of paralogous genes by the basal eukaryotes, and recently discovered correlation between polyploidy and the presence of histones in Archaea.

**Reviewer:**

This article was reviewed by Eugene Koonin, Uri Gophna and Armen Mulkidjanian. For the full reviews, please go to the Reviewers' comments section.

## Background

### Archaeal mating system and the origin of eukaryotic sex

Eukaryotic sex, or amphimixis (i.e., the presence of syngamy and meiosis in the life cycle), is characteristic of the vast majority of eukaryotes. The origin of amphimixis remains disputable. Several authors have postulated the existence of ancestral apomictic eukaryotes [1–3]. However, there is growing evidence that apomictic eukaryotic clades are descended from amphimictic ancestors, and that the evolution of sex was tightly linked to the emergence of other major eukaryote traits. Thus it is possible that some early forms of amphimixis already existed in the basal eukaryotes and probably even in their prokaryotic ancestors [4–8].

From this standpoint, it is interesting to look for possible intermediate forms of sex in extant Archaea. By now, the most promising case is the genetic transfer system of the halophilic archaeon *Haloferax volcanii* (Euryarchaeota: Halobacteriales). *Haloferax* is capable of genetic transfer which involves the formation of multiple cytoplasmic bridges between cells (and even the networks of interconnected cells), the transfer of plasmids and genomic DNA, and the capability of each cell to be both the donor and the recipient of the genetic material [9, 10]. Cell fusion can occur under laboratory conditions when cytoplasmic bridges become destabilized (this can be achieved by lowering the Mg^2+^ concentration to remove the cell envelopes [9]), and probably in nature [10].

Gross and Bhattacharya [4] have recently reviewed the molecular parallels between eukaryotic meiosis and archaeal genetic transfer system and suggested a plausible and detailed scenario for gradual evolutionary transition from *Haloferax*-type mating system to amphimixis. One important detail that has not received due attention in this context is the fact that *H. volcanii* is highly polyploid, with 17 (on average) copies of genome per cell during the exponential phase and 10 during the stationary phase [11, 12]. Many other Archaea are also polyploid (see below). This fact casts some doubt on the idea that eukaryotic sex originally evolved to promote double strand DNA break repair by homologous recombination [4, 13, 14], because a polyploid cell has enough genome copies of its own and does not need foreign DNA for this purpose.

Theoretically, polyploidy in prokaryotes can have interesting genetic and evolutionary outcomes, especially if the mechanisms of chromosome distribution during cell division are not very accurate and precise (i.e., there is no mitosis). In the current paper, we explore the possibility that polyploidy in the ancestral proto-eukaryotes could play a role in the origin of eukaryotic sex. This possibility comes from four hypothetical considerations: (1) proto-eukaryotes probably experienced an elevated risk of mutational degradation due to highly mutagenic environments of the shallow water habitats during the Great Oxygenation Event [4], invasion of type II self-splicing introns [15], and the supposedly rapid increase of gene complement and functional genome size during the eukaryogenesis [8, 16]; (2) under certain conditions, sex is an effective way to diminish the rate of accumulation of deleterious mutations [17, 18]; (3) the risk of genetic degradation may be higher in polyploid amitotic prokaryotes compared to monoploids (as discussed below), so that the former probably were under stronger selective pressure to develop some mechanisms analogous to eukaryotic sex (or some of its components) prior to, or in the course of, eukaryogenesis; (4) genetic redundancy of polyploids results in negative epistasis between beneficial alleles (i.e., recessive deleterious mutations have little effect until many or all copies of a gene are damaged, at which point fitness declines rapidly), and negative epistasis is known to enhance the ability of sex to improve the efficiency of selection against deleterious alleles [17, 18].

### Polyploidy in prokaryotes: costs and benefits

Prokaryotes are commonly assumed to possess only one circular chromosome per cell, and in fact many prokaryotes are monoploid. However, recent research has shown that polyploidy is common among Bacteria and Archaea [11]. Specifically, polyploidy appears to be a characteristic feature of the majority of clades within Euryarchaeota (e.g., Halobacteriales, Methanosarcinales, Thermococcales, and Methanococcales). Many Euryarchaeota have from 10 to 50 genome copies per cell in the exponential phase and about two times less in the stationary phase, while Crenarchaeota are typically monoploid [12, 19, 20].

The possible advantages of polyploidy in prokaryotes include efficient double-strand DNA break repair by homologous recombination ([11, 21], but see: [22]), higher rate of protein synthesis in restrictive environments, restrained phenotypic expression of deleterious recessive mutations, and the usage of redundant genomic DNA as phosphate storage polymer [23].

There are also costs to polyploidy, one of the most important ones being the risk of accumulation of deleterious recessive (or partially recessive) alleles. The evolutionary outcome here depends strongly on the way in which genome copies are segregated during cell division. Monoploid Bacteria and Archaea, as well as many plasmids, possess effective mechanisms (functionally analogous to eukaryotic mitosis) of accurate DNA segregation during cell division [24–26]. However, there is no evidence for such mechanisms (or tightly regulated cell cycle whatsoever) in polyploid Archaea. Halophilic Archaea and other polyploid Euryarchaeota probably rely on more or less stochastic chromosome distribution, which is sufficient to ensure that each daughter cell will have enough chromosomes to survive provided that the parent’s genome copy number is high [11, 27, 28]. The same is true for some high-copy-number plasmids that lack copy number control [29]. In some polyploid Archaea, however, the genome copy numbers appear to be more or less tightly regulated [12], although there are apparently no mechanisms capable of supplying each daughter cell with exactly one copy of each parental chromosome. The same is probably true for the mechanisms of mitochondria distribution during eukaryotic cell division [30].

The supposed absence of accurate and precise chromosome distribution (mitosis or its analogues) implies that polyploid Archaea may be prone to accumulation of segregation load. This means that viable cells will often produce unviable offspring. For instance, a polyploid cell with one intact copy of each of the several essential genes, but with other copies damaged by mutations, may be perfectly viable, but its chances to produce viable offspring may be negligible provided that intact genes are scattered over different chromosomes, and that each descendant gets a random set of chromosomes during cell division.

Moreover, amitotic polyploids are less likely, in the long term, to benefit from their genetic redundancy in the way eukaryotes sometimes do after whole-genome duplications. In amitotic polyploids, it is difficult for the two copies of a gene located on different copies of the chromosome to acquire different functions because there is no mechanism to ensure their stable joint inheritance. In the lab, this obstacle can be partially overcome by ongoing strong selection against homozygotes [12, 31], although such selection tends to become exceedingly wasteful with increasing number of heterozygous loci under selection. Modeling implies that partial specialization of genome copies is possible in amitotic polyploids, but only at the cost of high segregation load (see below).

It has been suggested that polyploid, amitotic, asexual prokaryotes might be so prone to genetic degradation via Muller’s ratchet (operating at the level of individual chromosomes rather than the whole polyploid genomes, which are not maintained during cell division) that they probably could not exist at all unless they developed special adaptations to escape the irreversible accumulation of deleterious recessive alleles [12]. Two such adaptations have been discussed in the context of polyploid Archaea: high-frequency lateral gene transfer (LGT) between genetically similar (conspecific) cells followed by homologous recombination [32] and equalization of the genome copies via gene conversion [12].

There is robust experimental evidence that halophilic Archaea employ both strategies extensively. For instance, natural populations of *Halorubrum* exchange genes so frequently that the degree of linkage equilibrium in their genomes approaches that of sexual populations [32]. Two species of *Haloferax, H. volcanii* and *H. mediterranei*, whose genomes have sequence identity of 86.6 %, are capable of interspecific genetic exchange which results in hybrids of the parent species. Whole genome sequencing revealed the exchange of DNA fragments up to 530Kb long (13 % of the genome) [33]. Gene conversion (i.e., asymmetrical homologous recombination resulting in one allele “overwriting” another) is used by methanogenic and halophilic Archaea to equalize genome copies, so that heterozygotes rapidly disappear from the population in the absence of selection [12, 31]. Interestingly, plant plastids, which are polyploid and retain many physiological and genetic features of their cyanobacterial ancestors, also use gene conversion to equalize genome copies, presumably in order to escape Muller’s ratchet [34].

There are at least two other conceivable strategies which polyploid prokaryotes can employ in order to slow down the accumulation of deleterious alleles. First, asexual ploidy cycles may help to lessen the mutation load by periodically producing monoploid cells and thus exposing recessive alleles to selection [35]. Second, evolution of accurate and precise segregation of sister chromosomes during cell division (mitosis) would remove all segregation load in asexual polyploids, making it possible for a cell with many of the redundant gene copies damaged by mutations to produce only viable offspring.

Here, we investigate the short-term evolutionary consequences of polyploidy in prokaryotes by means of computer simulation. We explore the efficiency of several possible strategies (and their combinations) to improve the evolutionary potential of polyploid prokaryotes and to minimize the risk of genetic degradation. We note that virtually all conceivable adaptations of this kind appear to be either essential components or pre-requisites for the eventual evolution of eukaryotic sex. We proceed to hypothesize, in line with the previous work [4, 36], that the initial stages of eukaryogenesis were stimulated by highly mutagenic conditions of the Early Proterozoic shallow-water habitats at the onset of the Great Oxygenation Event, and that some polyploid Archaea striving to survive in these restrictive conditions eventually developed mitosis, which is the most radical way to lessen the genetic costs of polyploidy. We further speculate that the emergence of mitosis in initially amitotic polyploid Archaea results in chromosome diversification, which, in turn, comes into conflict with the previously evolved modes of LGT and promiscuous recombination of chromosomes. The evolution of syngamy and meiosis appears to be a logical way to resolve this conflict.

## Methods

We devised a computer model that simulates evolution of a finite population of unicellular organisms. The model was designed to simulate different modes of genetic exchange, recombination, and chromosome distribution during cell division, and to allow for clear distinction between short-term evolutionary costs and benefits of such traits (and their combinations). In our model, evolutionary outcome of a trait is revealed by the dynamics of average fitness or ‘gene quality’ of the model population, which can demonstrate, e.g., slowing growth, random fluctuations around the average, steady decline, initial growth followed by decline, or vice versa, depending on the parameters. For technical information about the computer program, see “Availability of materials” section.

There are initially ***N*** individuals in the model population, and this number cannot be exceeded in the later generations. Each cell has ***P*** initially identical circular chromosomes (genome copies). Each chromosome contains ***G*** loci (genes). Each gene is characterized by ‘performance quality’ ***f***_***g***_ which ranges from 0 to 1 and can go down due to deleterious mutations or increase due to beneficial mutations. Initially, all genes have equal ***f***_***g***_ = ***f***_***0***_. Competitive ability (‘potential fitness’) ***F*** of a monoploid cell is calculated as a product of normalized ***f***s of all its genes: ***F*** = (***f***_***g1***_/***f***_***0***_)•(***f***_***g2***_/***f***_***0***_) •…• (***f***_***gG***_/***f***_***0***_). Therefore, a 5 % decrease in performance of any gene leads to 5 % decrease in the competitive ability of the individual. For a polyploid cell, the best copy of each gene is used to calculate ***F.*** This means that all beneficial alleles are dominant, all deleterious alleles are recessive. Initially ***F*** = 1 for all cells in the population. Cells with ***F*** < 0.5 are considered unviable and are excluded permanently from the population at the beginning of each step (generation).

In each step, cells reproduce by binary fission (producing 2 ***N*** individuals) and then undergo selection (selective survival). Survival of a cell in each generation depends on its ‘effective fitness’, ***F***_***e***_, which, in turn, depends on the combination of its relative ***F*** and random chance. The extent to which random chance influences survival is determined by parameter ***S***:

$$ Fe=\left(1-S\right)\bullet \left(F-{F}_{\boldsymbol{min}}\right)/\left({F}_{\boldsymbol{max}}-{F}_{\boldsymbol{min}}\right)+S\bullet Rnd, $$

where ***F***_***min***_, ***F***_***max***_ – minimum and maximum ***F*** in the population, ***Rnd*** – random fraction from 0 to 1. ***N*** cells with the highest ***F***_***e***_ survive, others die. Thus, the survival of a cell depends on its ***F*** relative to ***F***s of other cells in the population, except that cells with ***F*** < 0.5 are eliminated independently of their position in the ranking. This lower limit of ***F*** makes it possible for the population to decrease below ***N*** and eventually die out.

Parameter ***S*** can be used to regulate the intensity of drift. If ***S*** = 0, there is no drift, and the ‘best’ half of the population (that is, ***N*** cells with the highest ***F***s) invariably survives (unless some cells of the best half have ***F*** < 0.5). If 0 < ***S*** < 0.5, there is some drift, but there is still some portion of individuals with the highest ***F***s that cannot be eliminated (and therefore Muller’s ratchet cannot operate on individual level), and some portion of individuals with the lowest ***F***s that cannot survive. If ***S*** < 0.5, the behavior of the model is almost independent of ***N***, and the results obtained for small populations can be extrapolated to larger ones. If ***S*** > 0.5, drift is strong, and the evolutionary fate of the population depends strongly on ***N***.

This way of modeling selection imitates intense within-population competition for resources. The survival of the individual depends on its relative, rather than absolute, ‘genome quality’. If ***S*** < 0.5, there is negative epistasis between beneficial alleles (extreme case of such epistasis, truncation selection, is modeled when ***S*** = 0), which makes sex beneficial in the long term even in an infinite population [17, 18, 37].

In each generation, each gene mutates with probability ***M*** (mutation rate per chromosome ***U***_***chr***_ = ***M•G***; mutation rate per polyploid genome ***U***_***genome***_ = ***M•G•P***). Mutations are either deleterious or beneficial; the ratio is specified by parameter ***B*** (deleterious mutation rate: ***M***•(1-***B***); beneficial mutation rate: ***M***•***B***). Neutral mutations are irrelevant for our purposes, so we do not model them. Detrimental effect of deleterious mutations is regulated by parameter ***K***_***d***_: ***f***_***g new***_ = ***f***_***g old***_•(1 – ***K***_***d***_); positive effect of beneficial mutations is specified by parameter ***K***_***b***_: ***f***_***g new***_ = 1 – (1 – ***f***_***g old***_)•(1 – ***K***_***b***_). Thus, all deleterious mutations are equally deleterious, and all beneficial mutations are equally beneficial; our experiments with variable mutation effects revealed no substantial differences (data not shown).

Before cell division, in the simplest (default) case of constant ploidy, each chromosome is replicated, the order of the resulting 2***P*** chromosomes is randomly shuffled, and then one of the two daughter cells gets the first ***P*** chromosomes, and the other gets the remaining ones. If changes of ploidy are allowed, then the probabilities of unequal chromosome distribution (***Q***) and cell division without prior chromosome replication (***D***) are specified. In the former case, after chromosome replication one daughter cell receives ***P*** + 1 chromosomes (but not more than ***P***_***max***_, the upper limit of ploidy under conditions of variable ploidy), and the other receives ***P***-1 chromosomes (but not less than 1). In the latter case, each daughter cell receives either ***P***/2 chromosomes (when parental ploidy is even), or (***P*** + 1)/2 and (***P***-1)/2 chromosomes (when parental ploidy is odd). If mitosis is switched on, each descendant receives one replica of each parental chromosome. Mitosis is mutually exclusive with changes of ploidy.

The model allows two modes of within-individual recombination in polyploid cells: ‘gene conversion’ and ‘crossing over’. Gene conversion is specified by parameter ***R***_***conv***_ (conversion ratio). In each generation, ***P***•***N***•***R***_***conv***_ instances of gene conversion occur. During every such event, a length of a randomly chosen chromosome of a randomly chosen individual that contains 1 + ***G***/20 + Ri(***G***/10 + 1) adjacent genes is replaced by a copy of the homologous portion of another randomly chosen chromosome of the same individual (where Ri(X) is a random integer, 0 ≤ Ri(X) < X). Crossing over (parameter ***R***_***cross***_) is simulated similarly, except that the portions of chromosomes are swapped.

The model also allows three modes of between-individual genetic exchange: ‘lateral gene transfer’, ‘reciprocal chromosome exchange’ and ‘reciprocal chromosome exchange coupled with crossing over’. All three processes are affected by parameter ***homology***, which can be either switched on or off (true or false). When ***homology*** = false, random chromosomes of the two individuals are chosen. When ***homology*** = true, the first chromosome is randomly chosen from the genome of the first cell, while the second one is the chromosome of the second cell which is the most similar (contains the maximum number of identical genes) to the first chromosome. In the experiments described below, homology is switched off unless explicitly stated otherwise.

LGT is specified by parameter ***R***_***lgt***_ and is simulated in the same way as gene conversion, except that the two participating chromosomes belong to two different, randomly selected individuals. This corresponds to acquisition of foreign DNA via conjugation or natural transformation, followed by homologous recombination which results in the replacement of a chromosome fragment by the homologous stretch of foreign DNA. This mode of LGT has been shown to have evolutionary consequences similar to those of eukaryotic sex [8, 38].

Reciprocal chromosome exchange is specified by parameter ***R***_***ex***_. In each generation, ***P•N•R***_***ex***_ instances of chromosome exchange occur. During chromosome exchange, two selected chromosomes of two different, randomly selected individuals swap places.

Reciprocal chromosome exchange coupled with crossing over is specified by parameter ***R***_***pair***_. It is simulated as reciprocal chromosome exchange followed by crossing over between the two participating chromosomes. Chromosomes are chosen as specified above. In each generation, ***P•N•R***_***pair***_ instances of chromosome exchange with crossing over occur.

In the results discussed below, we assume complete dominance of beneficial alleles. In the case of incomplete dominance (when a polyploid cell heterozygous for a deleterious allele has lower ***F*** than a cell homozygous for the beneficial allele) the results of modeling are rather trivial. In this case, polyploidy is equivalent to having larger genome and higher ***U***_***genome***_. Therefore, if the maximum value of ***M*** a monoploid can tolerate is ***M***_***1***_, then the highest ***M*** acceptable for a polyploid will be approximately ***M***_***1***_***/P*** (data not shown). In this case, the evolutionary outcomes of polyploidy are not particularly interesting. It is for this reason, as well as for simplicity, that we restricted our analysis only to those genes of which a single intact copy is enough to ensure the survival of the cell.

We used the same ‘default’ set of parameters in most experiments (***N*** = 4000, ***f***_***0***_ = 0.99, ***G*** = 100, ***M*** = 0.007, ***B*** = 0.01, ***K***_***d***_ = 0.05, ***K***_***b***_ = 0.1, ***S*** = 0.3). This combination of parameters corresponds to a small local population, high mutation rate, reasonably low chances of beneficial mutations (the latter, in fact, may be quite frequent in microbial populations in novel environments [39]), and intense within-population competition (e.g., for resources) which results in negative epistasis between beneficial alleles [18], highly efficient selection, weak genetic drift, and prominent benefits of sex. Additional experiments have shown that general trends demonstrated by the model are essentially the same within wide spectrum of parameter combinations, although the details may differ. It is beyond the scope of this paper to reconstruct the precise conditions of some particular pre-Cambrian microbial communities; we use the model primarily to illustrate the theoretical possibility of the effects discussed.

## Results

### Polyploidy in prokaryotes increases the risk of genetic degradation and extinction

**Fig. 1:**
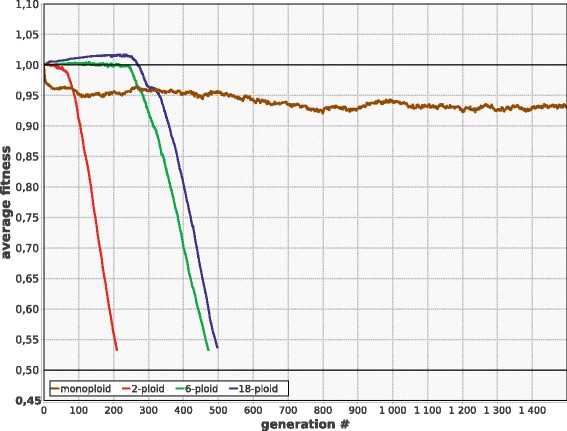
Average ***F*** in model populations with different ploidy (***P***). Parameters: ***N*** = 4000, ***f***_***0***_ = 0.99, ***G*** = 100, ***M*** = 0.007, ***B*** = 0.01, ***K***_***d***_ = 0.05, ***K***_***b***_ = 0.1, ***S*** = 0.3, constant ploidy, no LGT, no recombination

Unless the mutation rate (***M***) is low enough to ensure stable existence of the population regardless of ploidy, monoploids are more viable than polyploids in the long term. However, monoploids are at disadvantage in the short term (e.g., during the first few hundreds of generations). For instance, when ***M*** is moderately high (***M*** = 0.007, ***G*** = 100, ***U***_***chr***_ = 0.7), monoploid population reaches a stable mutation-selection balance and survives indefinitely, while polyploid populations demonstrate increased fitness during the first few hundred generations, but then rapidly degenerate and die out (Fig. 1).

The short term advantage of polyploids is due to: (1) delayed phenotypic expression of deleterious recessive alleles that accumulate freely while their frequency is low enough to prevent the production of homozygous offspring, and (2) increased beneficial mutation rate per genome, which equals ***G•M•B*** in monoploids and ***G•M•B•P*** in polyploids.

The eventual degeneration is due to lower efficiency of selection against deleterious alleles which results in accumulation of high segregation load. Also important is the fact that in the polyploid population the best remaining chromosome can easily be lost to drift (Muller’s ratchet at the level of individual chromosomes), because its bearers can have the same or even lower viability (***F***) than the cells with worse chromosomes in luckier combination. In monoploids, the best chromosome cannot be lost to drift (Muller’s ratchet does not operate) when ***S*** < 0.5.

**Fig. 2:**
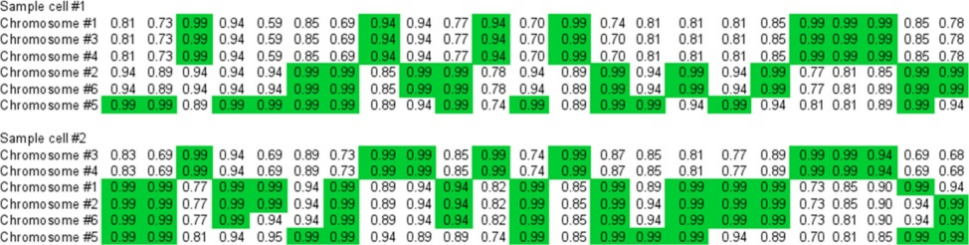
Genomes of two randomly chosen cells from the 500-th generation of a 6-ploid population (parameters as in Fig. 1). Numbers represent ***f***_***g***_s (note that the initial ‘quality’ of each gene ***f***_***0***_ equals 0.99); columns represent genes (the first 23 out of 100 genes are shown). For clarity, chromosomes are sorted based on their similarity to each other. Best alleles in each locus are shaded. Note that cells tend to possess two or three types of complementarily damaged chromosomes with different sets of (almost) intact genes; each chromosome type is represented by 1–3 copies

Modeling shows that polyploids do tend to lose their best chromosomes (with most genes intact), and survive henceforward due to quasi-stable retention of different combinations of complementarily damaged chromosomes. Usually there are a few types of such chromosomes in each cell, with a more or less unique set of damaged and intact genes in each type (Fig. 2).

Such cells may have high ***F*** and be perfectly viable, but their segregation load (probability of producing unviable offspring) is also high. This is because at least one chromosome of each type must get into each daughter cell in order to ensure that fitness of the offspring will be not much lower than that of the parent. The probability of such lucky chromosome distribution varies by ploidy level, number of chromosome types, and abundance of each chromosome type in the parent’s genome.

Populations with higher ploidy are generally better off than diploids and oligoploids due to several reasons. Firstly, effective population size (number of chromosomes per population) is higher in polyploid populations, provided that ***N*** is constant. Secondly, oligoploids are not as good as polyploids in accumulating rare beneficial mutations, while deleterious alleles start being expressed in their phenotype almost immediately. From the other hand, selection against deleterious alleles becomes weaker with increasing ploidy. The long term evolutionary outcome of polyploidy also depends on the typical number of types of complementarily damaged chromosomes that becomes established in the cell lineages in the course of evolution (Fig. 2), which depends on parameters, so that the resulting relationship between ploidy and resistance to extinction may be quite complicated.

**Fig 3:**
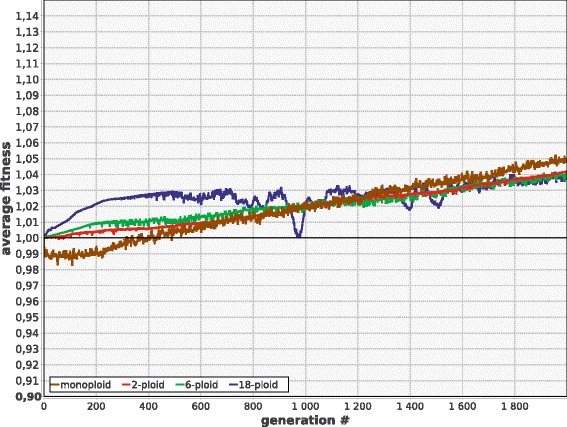
Monoploids demonstrate faster growth of ***F*** than polyploids at low mutation rate. ***M*** = 0.005; other parameters and designations as in Fig. 1

Importantly, even if ***M*** is low enough to ensure the survival of polyploids, monoploids still possess long term fitness advantage (Fig. 3).

### Polyploidy as an ‘evolutionary trap’

**Fig. 4:**
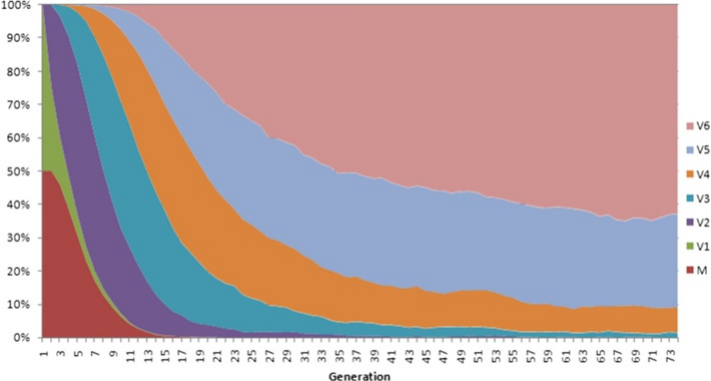
Proportion of cells with different ploidy in a population of cells capable of unequal chromosome distribution during cell division. Cells with the highest ploidy outcompete all the others. Average ploidy hence approaches the allowed maximum (in this case, ***P***_***max***_ = 6), even if this eventually results in extinction. In this case, the population initially consists of 2000 obligate monoploids (M) and 2000 cells of variable ploidy. The latter are initially monoploid (V1). Occasionally (with probability ***Q*** = 0.2) they produce offspring with one extra chromosome (V2 … V6); if parent’s ploidy ***P*** > 1, one of its offspring with 20 % probability gets ***P***-1 chromosomes, and the other gets **P** + 1 (see ‘Description of the model’ for more technical details). All other parameters as in Fig. 1. This model population died out after 677 generations, although it would survive indefinitely if changes of ploidy were not allowed and all cells remained monoploid

The results described in the previous section imply that polyploidy may present an evolutionary trap, first ‘luring’ the population by the short term advantage, and then driving it to extinction. Indeed, this is what happens with the model populations when ploidy is allowed to fluctuate occasionally, ***M*** is low enough for monoploids to survive, but too high for polyploids (Fig. 4). In this case, polyploid cells quickly outcompete those with lower ploidy, drive the obligate monoploids to extinction despite their superior long term evolvability, and then proceed to accumulate genetic load and eventually die out.

This happens even if the initial proportion of cells capable of unequal chromosome distribution is very small (data not shown). Polyploidy spreads like infection and drives the population to extinction.

These results imply that polyploidy is a risky evolutionary strategy, and that stable existence of polyploid prokaryotes, especially in highly mutagenic environment, requires special adaptations to improve evolvability and diminish the risk of irreversible accumulation of genetic load. Such adaptations, even if not beneficial at the individual level, may evolve via group selection or second-order selection for evolvability [40].

We explored the efficiency of four conceivable types of such adaptations.

### Ploidy cycles as a way to lessen the mutation load

**Fig. 5:**
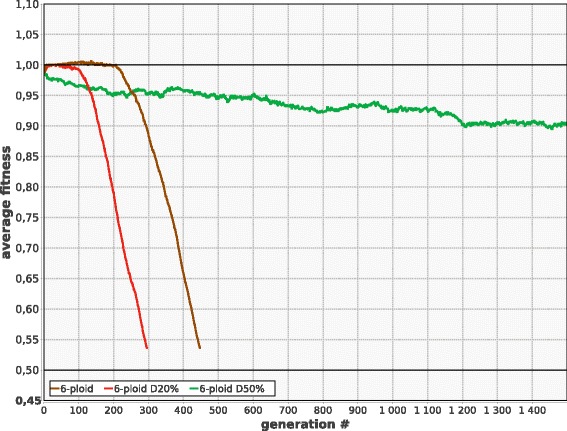
Ploidy cycles can be detrimental when rare, but beneficial when frequent. Population of initially monoploid cells with variable ploidy, ***P***_***max***_ = 6, without reductional divisions (***D*** = 0), was dominated by hexaploids and died out after 440 generations (6-ploid). With 20 % reductional divisions (***D*** = 0.2, 6-ploid_D20%), the same population died out after 290 generations, because the population was dominated by diploids, and diploids are more prone to extinction than hexaploids (see Fig. 1). However, more frequent reductional divisions (***D*** = 0.5, 6-ploid_D50%) rescued the population, because in this case a substantial proportion (35-40 %) of cells in each generation were monoploid. Other parameters as in Fig. 1

Asexual ploidy cycle is the periodic production of offspring with lower ploidy, e.g., by means of cell division without prior chromosome replication or destruction of redundant chromosomes, coupled with periodic increases of ploidy by means of more frequent replication or less frequent cell divisions. Ploidy cycles have been shown to lessen the mutation load in unicellular asexual (apomictic) eukaryotes by producing monoploid cells and thus exposing recessive alleles to selection [35]. Our model shows that ploidy cycles may have similar beneficial effect in polyploid amitotic prokaryotes, but only if the reductional divisions are frequent enough to ensure the presence of substantial proportion of monoploid cells in the population. Rare reductional divisions can be useless or even detrimental. For instance, if the upper limit of ploidy in the population of cells with variable ploidy ***P***_***max***_ = 6, and the probability of reduction ***D*** = 0.2, then diploids and triploids, rather than monoploids, will prevail in the population. Consequently, the population will degenerate and die out even faster than without reductional divisions, because in the latter case the population will be dominated by hexaploids which are generally better off than diploids. Another reason why reductional divisions can be detrimental is that they promote the disruption of the lucky combinations of partially damaged chromosomes which often form the basis for the long term survival of polyploid populations. However, if ***D*** is high enough to produce numerous monoploids in each generation, the population can be rescued (Fig. 5). Production of monoploids is essential for the beneficial effect of the ploidy cycle because the presence of monoploids allows purifying selection to act efficiently against recessive deleterious alleles.

### Equalization of genome copies by gene conversion as a way to lessen the mutation load

In polyploid Euryarchaeota and plant plastids, frequent gene conversion (asymmetrical recombination of homologous portions of different copies of the chromosome) results in effective equalization of genome copies and rapid disappearance of heterozygotes from the population even in the absence of selection [12, 31, 34]. Presumably, this mechanism allows asexual, amitotic polyploids to escape Muller’s ratchet, because gene conversion makes it possible to produce a functional chromosome from two damaged ones [31, 41].

**Fig. 6:**
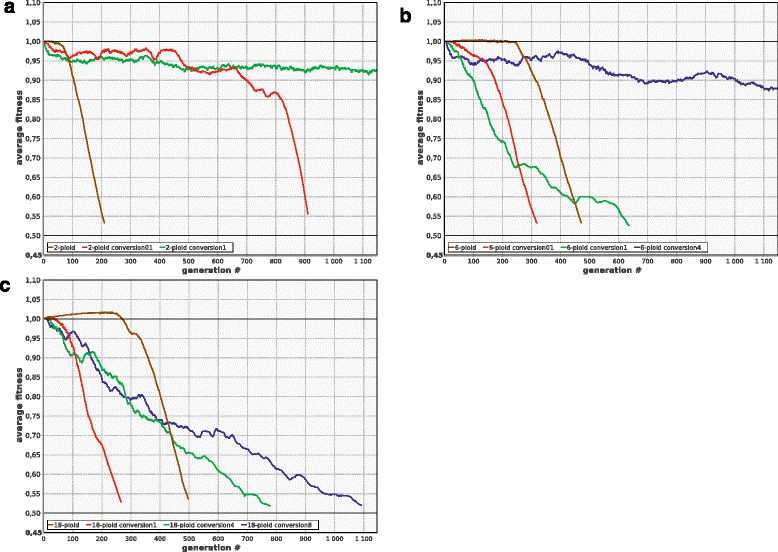
Gene conversion rescues oligoploid populations, but can be detrimental if ploidy level is high and conversion is not frequent enough to ensure homozygosity. **a** In diploid population, even infrequent gene conversion is beneficial. 2-ploid_conversion01: ***R***_***conv***_ = 0.1, each gene undergoes conversion with average probability 0.011 per generation, which is comparable with mutation rate (M = 0.007); 2-ploid_conversion1: ***R***_***conv***_ = 1, probability of conversion is 0.11 per gene, which is 1 order of magnitude higher than the mutation rate; the population is rescued. **b** In 6-ploid population, conversion rates ***R***_***conv***_ = 0.1 and 1 are detrimental, and only at ***R***_***conv***_ = 4 (conversion probability 0.44, which is 63 times higher than the mutation rate) the population is rescued. **c** In 18-ploid population, even ***R***_***conv***_ = 8 (conversion rate 0.88) is not enough to rescue the population

Our model suggests that equalization of genome copies by gene conversion can rescue polyploid populations, but only if this process is frequent enough to radically increase homozygosity. This is relatively easy to do in di- and oligoploid populations, but extremely difficult if ploidy level is high. If gene conversion fails to produce high levels of homozygosity, it can be useless or even detrimental (Fig. 6).

The positive effect of gene conversion comes from bringing alleles into homozygous state and thus exposing them to selection, as well as from the possibility of construction of a better chromosome from two damaged ones, so that Muller’s ratchet can be avoided. Its negative effect is due to the destruction of beneficial combinations of partially damaged, complementary chromosomes (Fig. 2). The latter statement requires explanation.

Imagine a viable (fitness ***F*** = 1) triploid cell with the following chromosomes: (1) 1100101, (2) 1100101, (3) 0011010, where 1 s represent functional loci and 0 s represent recessive lethals. Modeling suggests that such cells with several copies of each of a few types of complementarily damaged chromosomes tend to prevail in polyploid populations (Fig. 2). After chromosome replication, the genome of the cell will be: 11’22’33’. When the cell divides, one of the following ten pairs of offspring can be produced with equal probability: 11’2 + 2’33’, 11’2’ + 233’, 11’3 + 22’3’, 11’3’ + 22’3, 122’ + 1’33’, 123 + 1’2’3’, 123’ + 1’2’3, 12’3 + 1’23’, 12’3’ + 1’23, 133’ +1’22’. Of these 20 potential offspring, 16 are viable and 4 are not (segregation load 4/20 = 0.2).

Now imagine that gene conversion occurred in the parent cell, so that an allele from chromosome 3 was copied to the homologous position on chromosome 2 (or vice versa). If the copied allele is functional, the recipient chromosome may improve, but the segregation load will not become smaller unless *all* deleterious alleles on the chromosome are replaced by functional ones, which is unlikely. For instance, for the genome 1100101, 11001***1***1, 0011010 (one locus on chromosome 2 was improved by gene conversion), segregation load is still 0.2 (fitness also remains the same: ***F*** = 1). However, if the copied allele is deleterious, segregation load will increase. For instance, for the genome 1100101, 1100***0***01, 0011010 (one locus on chromosome 2 was damaged by gene conversion), segregation load is doubled (0.4). If a lethal recessive allele is copied to the ‘unique’ (non-redundant) chromosome 3, this will result in immediate cell death. This example shows that it is difficult for gene conversion to lessen segregation load of a polyploid cell with many deleterious recessive alleles, and very easy to inflate it. However, if gene conversion rate is much higher than mutation rate, new mutations will be either quickly ‘overwritten’, or brought into homozygous state and thus exposed to selection.

Symmetrical reciprocal recombination (crossing over) provides no substantial benefits and is often deleterious for polyploids (e.g., with parameters listed in Fig. 6, symmetrical recombination, if used instead of gene conversion, does not rescue the model populations and is deleterious in most cases). This is because crossing over cannot bring alleles into homozygous state and expose them to selection, although it efficiently destroys quasi-stable combinations of complementarily damaged chromosomes.

### Lateral gene transfer followed by homologous recombination as a way to lessen the mutation load

**Fig. 7:**
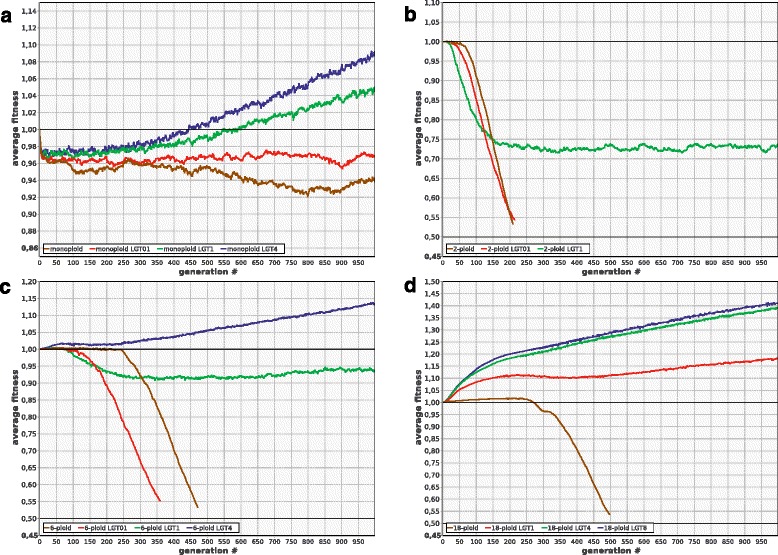
LGT improves evolvability and rescues polyploid populations from extinction. **a** monoploids (1-ploid: no LGT (***R***_***lgt***_ = 0), 1-ploid-lgt01: ***R***_***lgt***_ = 0.1, 1-ploid-lgt1: ***R***_***lgt***_ = 1). **b**-**d** 2-, 6-, and 18-ploids; other parameters and designations as in Fig. 6 **a**-**c** except that LGT is modeled instead of gene conversion

In our model, LGT is simulated as the replacement of a portion of a chromosome by a copy of the homologous portion of other microbe’s chromosome [38]. Modeling implies that LGT is an efficient way to improve evolvability and rescue populations that are otherwise doomed to extinction (Fig. 7). It appears to be a far more efficient remedy against genetic degradation than gene conversion (compare Figs. 6 and 7).

**Fig. 8:**
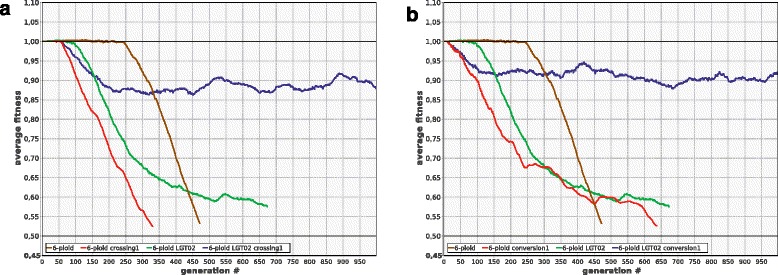
LGT acts synergistically with crossing over and gene conversion to enhance evolvability of polyploids. **a** In 6-ploids, moderately frequent crossing over (***R***_***cross***_ = 1) is detrimental (6-ploid-crossing1), and moderately frequent LGT (***R***_***lgt***_ = 0.2) is virtually useless (6-ploid-lgt02). In combination, however, these two processes rescue the population (6-ploid-lgt02-crossing1). The same is true for the combination of LGT and gene conversion **b** Other parameters and designations as in Fig. 1

Interestingly, crossing over, which is generally useless or deleterious without LGT (see above), acts synergistically with LGT, enhancing strongly its positive effect (Fig. 8a). The same is true for the combination ‘LGT + gene conversion’: even if both processes are not frequent enough to be useful by themselves, their combination can rescue the population (Fig. 8b). The synergistic effect of these combinations is apparently due to the fact that they enhance the production of better chromosomes from the fragments of the damaged ones and efficiently decouple positive selection of beneficial alleles from negative selection against deleterious ones by making recombination possible both between and within cells. The combination ‘crossing over + gene conversion’ does not have any synergistic effect (data not shown), apparently because both types of recombination operate within cells only.

### Chromosome exchange, coupled with recombination, helps to lessen the mutation load

**Fig. 9:**
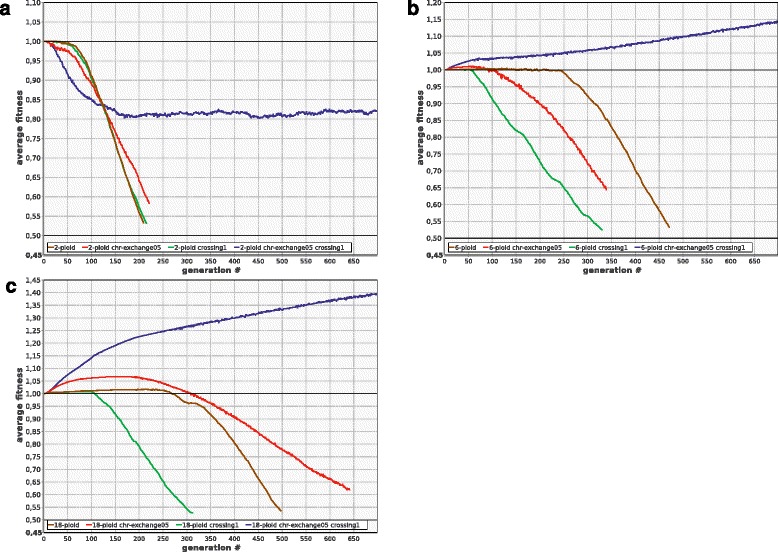
Chromosome exchange is useless by itself, but confers strong advantage when combined with crossing over. **a** 2-ploids (2-ploid: ***R***_***ex***_ = 0, ***R***_***cross***_ = 0; 2-ploid-chr-exchange05: ***R***_***ex***_ = 0.5, ***R***_***cross***_ = 0; 2-ploid-crossing1: ***R***_***ex***_ = 0, ***R***_***cross***_ = 1; 2-ploid-chr-exchange05-crossing1: ***R***_***ex***_ = 0.5, ***R***_***cross***_ = 1). **b** 6-ploids. **c** 18-ploids. Other parameters as in Fig. 1

It is not known whether halophilic polyploid Archaea can exchange complete chromosomes, but this seems plausible from what is known about their mating system [9, 10, 33]. We modelled reciprocal exchange of individual chromosomes (one randomly chosen chromosome of the first cell is replaced by a random chromosome of the second cell, and vice versa). We found that such exchange is virtually useless per se, but extremely advantageous when combined with the shuffling of chromosome fragments within the cell by means of either gene conversion or crossing over (Fig. 9).

### Mitosis as the most radical way to improve the evolvability of polyploids

**Fig. 10:**
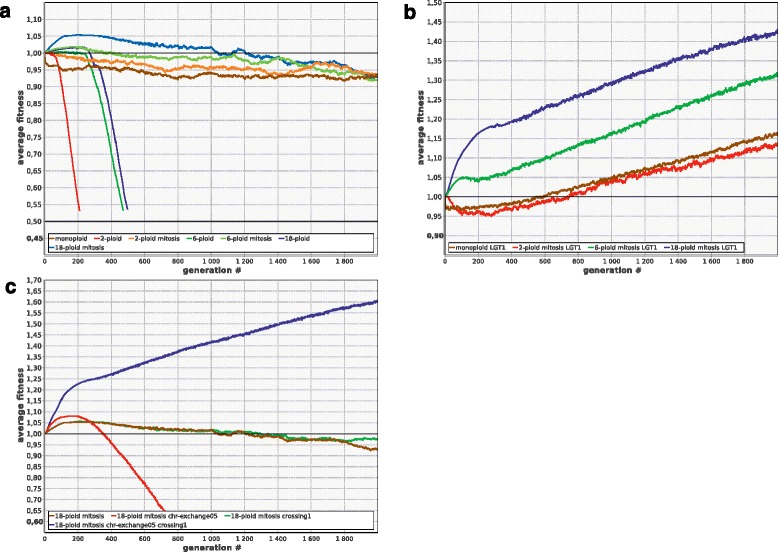
Evolutionary effects of mitosis in polyploids. **a** Mitosis removes disadvantages of polyploidy. **b** Positive effect of LGT is retained. **c** Crossing over and chromosome exchange are useless or deleterious by themselves, but highly beneficial in combination. Parameters and designations as in the previous figures

Modeling suggests that mitosis (accurate segregation of sister chromosomes) immediately removes all long-term disadvantages of polyploidy. Mitotic polyploids initially are better off than monoploids, but finally they converge to the same level of fitness as only one intact copy of each gene is left in their genomes (Fig. 10a). Moreover, mitotic polyploids retain all the benefits of LGT (Fig. 10b). Chromosome exchange and within-genome recombination (crossing over) in mitotic polyploids are useless or even deleterious by themselves, but provide strong advantage when combined (Fig. 10c). However, even this strong advantage is somewhat smaller compared to amitotic polyploids (compare Figs. 9c and 10c). This is because random DNA shuffling comes into conflict with the process of chromosome specialization and diversification which is characteristic of mitotic polyploids (see below).

### Acquisition of mitosis by polyploids leads to chromosome specialization and diversification

In mitotic polyploids, the problem of segregation load is totally removed, and thus the genome copies (chromosomes) become free to diversify. If each daughter cell reliably receives exactly one copy of each parental chromosome, then each chromosome is free to accumulate any number of deleterious recessive mutations as long as at least one intact copy of each gene is retained on any other chromosome. In the absence of DNA shuffling, this will inevitably lead to fast diversification and specialization of chromosomes, so that each chromosome will bear its own unique set of intact genes. Although our model does not allow sub-functionalization (‘division of labour’ among paralogs) or neo-functionalization (evolution of new functions) of the redundant gene copies, these processes would become highly plausible if a real amitotic, polyploid microbe acquired mitosis. In other words, acquisition of mitosis transforms an amitotic, polyploid cell into an effectively monoploid, multi-chromosome cell whose genetic material has undergone whole-genome amplification. Evolutionary consequences of such transformation may be quite remarkable (see ‘Discussion’).

**Fig. 11:**
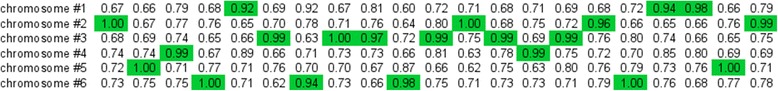
Genome of a randomly chosen cell from the 1000-th generation of a mitotic 6-ploid population (same as in Fig. 10a). Numbers represent ***f***_***g***_s; columns represent the first 23 genes out of 100 (***G*** = 100). Best alleles in each locus are shaded. Note that each chromosome has a unique set of well preserved (or improved, given that ***f***_***0***_ = 0.99) genes; each gene is well preserved only on one chromosome

Modeling confirms that the acquisition of mitosis leads to chromosome specialization (Fig. 11).

### Specialization of chromosomes encourages the evolution of homolog synapsis

As chromosomes become specialized, their random shuffling becomes less advantageous. In this case, selection may favour mechanisms that restrict homologous recombination only to the pairs of very similar chromosomes. The initial stages of the evolutionary transition to such “positive assortative mating” of the chromosomes may be simple, because homologous recombination is generally guided by homology. Moreover, some vestigial mechanisms of similarity-based chromosome pairing may evolve even in amitotic polyploids, as far as they also tend to possess several distinct chromosome types (Fig. 2). This can eventually result in the evolution of specialized mechanisms of homolog synapsis.

In order to illustrate the advantages of ‘choosy’ recombination between similar chromosomes in mitotic polyploids, we modelled the following situation: two randomly chosen cells meet, one random chromosome of the first cell and the most similar chromosome of the second cell are selected; the two chromosomes recombine by crossing over; recombinant chromosome of the first cell passes into the second cell and vice versa; cells separate (***R***_***pair***_ > 0, ***homology*** = true, see Methods).

**Fig. 12:**
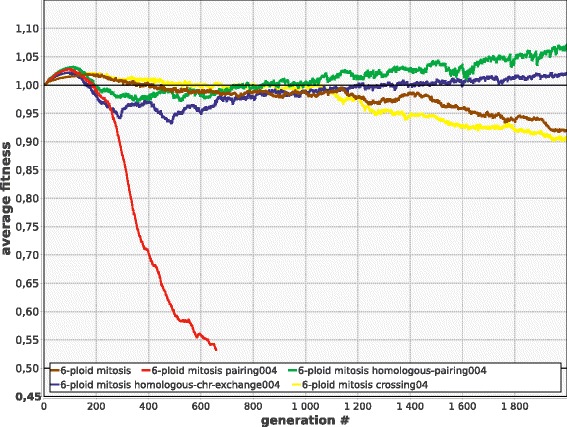
In mitotic polyploids, promiscuous (random) chromosome exchange coupled with crossing over may be deleterious (6-ploid mitosis pairing004: ***R***_***pair***_ = 0.04, ***homology*** = false), while the same process between similar chromosomes (6-ploid mitosis homologous-pairing004: ***R***_***pair***_ = 0.04, ***homology*** = true) confers long term advantage. Moreover, ‘choosy’ chromosome exchange provides some advantage even without crossing over (6-ploid mitosis homologous-chr-exchange004: ***R***_***ex***_ = 0.04, ***homology*** = true). Crossing over without chromosome exchange (6-ploid mitosis crossing04: ***R***_***cross***_ = 0.4) is neutral. Other parameters as in Fig. 10a (6-ploid mitosis)

We find that if the rate of chromosome exchange and recombination is high, then intense shuffling prohibits chromosome specialization. In this case it does not matter whether recombination is choosy or not, and promiscuous recombination confers advantage in the same way as it does in amitotic populations (as illustrated in Fig. 10c). However, if recombination is less frequent, chromosomes do specialize, and in this case it turns out that only choosy recombination is advantageous, while promiscuous recombination may be deleterious (Fig. 12). Apparently, this effect would be much more pronounced if our model allowed for the acquisition of new functions by the redundant gene copies.

## Discussion

Eukaryogenesis probably took place in the Early Proterozoic shallow water microbial communities, when O_2_ concentration started to rise, but the ozone layer has not yet formed, resulting in high reactive oxygen species concentrations [4]. This, together with other mutagenic factors such as the invasion of type II self-splicing introns from alphaproteobacteria that further gave rise to mitochondria, probably created the selective pressure that stimulated radical changes in the genetic architecture and genetic exchange strategy of proto-eukaryotes. Additional risk was provided by the increase in essential gene content and genome size which accompanied eukaryogenesis, because a larger genome (***G***) together with constant per-site mutation rate (***M***) also results in higher ***U***_***genome***_ (per-genome mutation rate).

We hypothesize that eukaryotes are descended from polyploid Archaea that gradually evolved a set of adaptations aimed to sustain the increased per-genome mutation rate. These adaptations eventually combined into what is known as eukaryotic sex.

The polyploidy itself, to begin with, may have been such adaptation, because the presence of additional genome copies is essential for repairing multiple double-strand DNA breaks (e.g., [21]). However, this was not necessarily so, because: (1) polyploidy also provides other benefits (e.g., [23]); (2) the correlation between ploidy and the ability to repair double strand DNA breaks is not absolute, e.g., *H. volcanii* is highly polyploid, but not particularly radioresistant [11]; (3) double-strand DNA break repair by homologous recombination may be tricky in a highly polyploid cell because each DNA end will have multiple partners [22].

From the other hand, polyploidy delays phenotypic expression of deleterious mutations, and thus can be selected for in highly mutagenic environment. Modeling suggests that polyploidy can confer strong short term competitive advantage, especially when mutation rate is high, because: (1) it restrains phenotypic expression of novel recessive deleterious mutations, (2) it accelerates accumulation of rare dominant beneficial mutations. Thus polyploidy can spread in the population despite its possible long-term deleterious effects.

We further suggest that polyploid proto-eukaryotes were capable of more or less precise control of their genome copy number, but initially had no mitosis-like mechanism to ensure that each daughter cell receives exactly one copy of each parental chromosome. This is possibly the case with the extant polyploid Euryarchaeota [11, 27]. In the absence of mitosis, polyploidy results in accumulation of segregation load. Modeling suggests that this is really the case in amitotic polyploid populations which are thus more prone to extinction at high mutation rates, compared to monoploids. Therefore, in order for polyploidy to retain its advantages, polyploid prokaryotes living in highly mutagenic environment have to develop some means of improving their evolvability and reducing their mutation load. In microbial populations, such adaptations can evolve, even if they are not immediately beneficial to the individual, by means of the so called ‘second order selection for evolvability’. The efficiency of this type of selection was demonstrated in the long term evolutionary experiment on *E. coli* [40].

There are several possible ways in which this goal can be achieved by amitotic polyploids, and, strikingly, all of them resemble different components or aspects of eukaryotic sex.

*The first possibility* is within-individual homologous recombination which can be either asymmetrical (gene conversion) or symmetrical (crossing over). Gene conversion may be useful because it ultimately brings alleles into homozygous state, thus exposing them to selection. Polyploid Archaea (as well as plant plastids) widely use gene conversion, presumably in order to equalize genome copies and escape genetic degradation via Muller’s ratchet [12, 20, 31, 34]. Modeling suggests that gene conversion, by itself, is only useful when very frequent; however, it is useful even when rare if combined with inter-individual genetic exchange. Crossing over, by itself, is useless for polyploids because it cannot provide homozygosity; however, it is very advantageous in combination with LGT or reciprocal exchange of chromosomes (see below).

*The second possibility* is asexual ploidy cycle which can periodically produce monoploid cells and thus expose alleles to selection [35]. However, modeling suggests that ploidy cycles can reduce genetic load only when reductional divisions are frequent enough to produce substantial proportion of monoploids. There is no indication that polyploid Archaea ever go that far, although they do have ploidy cycles: several species have been shown to reduce their ploidy approximately by half at the transition from exponential to stationary phase [11, 19]; they can also respond to phosphate availability by changing ploidy levels [23]. Apparently, these ploidy cycles have adaptive significance unrelated to the lessening of the mutation load.

*The third possibility* is high frequency LGT between relatives (conspecifics or members of closely related species). Polyploid Archaea use this option extensively [9, 10, 32, 33]. Incorporation of foreign genetic material acquired during mating which involves the formation of cytoplasmic bridges or temporary cell fusion [10] can be accomplished either by its insertion into the recipient’s chromosome in addition to the recipient’s own genes, or by homologous recombination which results in the replacement of the recipient’s alleles by the donor’s alleles. Apparently, only the second option (which is simulated in our model) can be used on the regular basis (e.g., in every generation) without undue expansion of the genome and disruption of the genetic architecture.

The positive effect of LGT on evolvability increases with LGT frequency. However, it has been shown that frequent LGT of the type we simulate in our model, which involves the replacement of the recipient’s alleles by foreign DNA fragments, can be evolutionary unstable. This is because modifier alleles that promote such recombination will tend to be ‘overwritten’ by competing alleles that prohibit recombination, while the latter will not be overwritten by other alleles exactly because they prohibit recombination. Consequently, modifier alleles that block LGT may behave as ‘selfish genes’ and spread in the gene pool despite the fact that LGT is beneficial for population and individuals [8]. This is probably one of the reasons why prokaryotic LGT cannot provide the intensity of inter-individual recombination comparable to that of eukaryotic sex (although it can be frequent enough to ensure comparable degree of linkage equilibrium [32]). Other reasons for the low frequency of promiscuous prokaryotic LGT include the risk of acquisition of incompatible genes, aggressive transposable elements and prophages [42].

It is theoretically possible for polyploid prokaryotes to circumvent this limitation by exchanging complete chromosomes (rather than chromosome fragments) and by shuffling them symmetrically (by crossing over) rather than asymmetrically (by gene conversion). In this case, no alleles become overwritten, all genes retain their chances to be passed to the next generation, and there are thus no preconditions for the spread of selfish modifiers.

It seems plausible, although not directly proven, that polyploid Euryarchaeota which are capable of forming cytoplasmic bridges between cells (or even of temporary cell fusion [10]) can exchange complete chromosomes. Modeling shows that such exchange can be highly advantageous when coupled with chromosome shuffling by means of either asymmetrical or symmetrical homologous recombination. Both mechanisms of homologous recombination are present in polyploid Archaea, although asymmetrical gene conversion seems to prevail [31, 43]. As noted above, the latter option (chromosome exchange + crossing over) is more evolutionary stable and can be used frequently without the danger of the spread of selfish modifier alleles that prohibit recombination.

Frequent crossing over would require linear, rather than circular chromosomes, because two circular chromosomes cannot segregate properly after recombination if the number of crossovers is odd [7, 44]. Interestingly, although the circular chromosome of *H. volcanii* contains four DNA replication origins, engineered strains in which all origins have been deleted grow even faster than wild type. These strains initiate replication at dispersed sites by means of homologous recombination and require the recombinase RadA for growth [28]. This means that replication initiation mechanisms in polyploid Archaea may be redundant and thus capable of rapid evolutionary change. Another implication is that the functions of homologous recombination in polyploid Archaea are quite diverse.

We hypothesize that ancestral proto-eukaryotes were amitotic polyploid Archaea that used a combination of strategies to improve their evolvability and reduce their genetic load in highly mutagenic environment. This combination included high-frequency LGT in the form of mating accompanied by the formation of cytoplasmic bridges (or temporary cell fusion), exchange (probably reciprocal) of complete chromosomes, symmetrical homologous recombination (crossing over) between chromosomes, and probably also ploidy cycles (periodical reductional divisions).

It must be stressed out that most of these strategies make sense for polyploids only, and are useless or hardly accessible for monoploids; and that all of them resemble (and are probably related to) different components or stages of eukaryotic sex. It should also be remembered that polyploids are basically more prone to extinction (due to accumulation of mutation load) than monoploids, and thus are more likely to develop additional adaptations of this kind, especially when mutation rate is high.

*The fourth* (and the most radical) strategy that polyploid amitotic prokaryotes may use in order to sustain high mutation rates is to develop mitosis, i.e., a mechanism of accurate chromosome segregation which ensures that each daughter cell receives exactly one copy of each individual chromosome of the parent. Such mechanisms (prokaryotic analogues of mitosis) exist in monoploid Bacteria and Archaea [25, 26], but are supposedly absent in polyploid Euryarchaeota who seem to rely on random segregation of numerous genome copies between daughter cells. Genome copy number, unlike the exact chromosome identities, may be more or less precisely controlled [11, 27, 28].

Acquisition of mitosis immediately removes all the risks related to the accumulation of segregation load. However, it retains, at least initially, the supposed advantages of polyploidy, including the short-term advantage which arise from the higher rate of accumulation of rare dominant beneficial mutations.

The inevitable consequence of this evolutionary innovation in asexual polyploids is more or less rapid chromosome diversification and specialization. If each chromosome of the parent is guaranteed to pass its copy into each daughter cell, then recessive deleterious mutations can accumulate freely (without creating any segregation load) as long as at least one copy of each gene, located on any chromosome, remains intact. Moreover, it is highly plausible that some (or many) of the redundant gene copies will acquire new functions (neo-functionalization), or that different copies of the gene will specialize on different aspects of the original function (sub-functionalization). Thus, acquisition of mitosis eventually transforms a polyploid cell into a functionally monoploid one with multiple unique, mutually irreplaceable, highly redundant chromosomes.

Between-individual chromosome exchange coupled with homologous recombination (crossing over) retains its positive effect in mitotic polyploid proto-eukaryotes, at least initially. So it seems unlikely that proto-eukaryotes would give up this useful mechanism after acquiring mitosis. Later, however, problems will arise, because random chromosome exchange and recombination will come into conflict with the ongoing process of chromosome diversification and specialization.

Initially, while all the chromosomes were similar and, to a large extent, mutually replaceable, it did not matter which (and how many) of them were swapped and shuffled in each round of between-individual genetic exchange. Later, as the chromosomes became more diverse and unique, and as the cells grew adapted to constant chromosome number, it became disadvantageous to exchange or shuffle them randomly. Some measures should have been taken in order to prevent the exchange of different (mutually irreplaceable) chromosomes, and to ensure that every chromosome takes part in between-individual genetic exchange with reasonable frequency.

We hypothesize that the acquisition of adaptations aimed to resolve the conflict between chromosome specialization (promoted by the acquisition of mitosis) and chromosome exchange and shuffling (inherited from amitotic ancestors) were the key to the evolution of eukaryotic sex.

If every chromosome is unique and essential for survival, then the cell cannot just pass one or two of them, randomly chosen, into another cell via cytoplasmic bridge and receive one or two foreign chromosomes, also randomly chosen, in return. This will result in unviable genome or, at best, in aneuploidy, which probably will also be disadvantageous, given that the cell is already adapted to constant chromosome content. Another problem with the exchange of individual chromosomes is that it cannot ensure that each chromosome will take part in recombination with optimal frequency. Apparently, the best and simplest way to solve these problems is to develop cell fusion (probably already present in the ancestral polyploid Archaea), to restrict recombination (crossing over) to the pairs of very similar (highly homologous) chromosomes, and to perform reductional division immediately after recombination, while the chromosomes are still in pairs and thus are ready to segregate accurately into the daughter cells. It is essential for such reductional division to ensure that each daughter cell receives exactly one chromosome from each homologous pair. Hence the already present mechanism of mitosis seems to be an excellent basis (pre-adaptation) for the development of such reductional division.

Indeed, molecular studies strongly suggest that meiosis evolved from mitosis [45, 46]. Wilkins and Holliday [3] have suggested a plausible and well-substantiated scenario of evolution of meiosis from mitosis, with which our ideas are highly compatible. They argued that the development of homologous chromosome pairing was the key initial event in the origin of meiosis, and that such pairing evolved not because it promotes intense recombination, but rather because it reduces the chances of ectopic pairing and consequent recombination of non-homologous (dissimilar) chromosomes. This is exactly what should have happened according to our idea that meiosis evolved in order to retain the benefits of between-individual recombination after the acquisition of mitosis and consequential specialization of chromosomes of the original amitotic polyploid ancestor. Indeed, our hypothesis provides explanation for the “*selection pressures to limit ectopic recombination and promote the accuracy of recombination*” suggested by Wilkins and Holliday [3].

Some primitive forms of homolog synapsis may have evolved prior to acquisition of mitosis, because the initial stages of chromosome specialization may have been already present in amitotic polyploids (Fig. 2). Choosy recombination, in its turn, promotes the evolution of mate choice, because it is dangerous to swap and shuffle chromosomes with a mate with different chromosome complement. This will ultimately lead to the emergence of “biological species” with well-mixed and reasonably isolated gene pools. Before the effective mechanisms of mate choice evolved, however, proto-eukaryotes may have acquired many new genes and gene complexes from distantly related lineages.

Additional support for the idea that eukaryotes evolved from polyploid Archaea comes from the recently discovered fact that polyploidy in Archaea appears to correlate strongly with the presence of histones. It seems plausible that histones play a role in enabling polyploidy in Archaea because they promote compact packaging of multiple genome copies in a single prokaryotic cell [19]. Indeed, the presence of histones in some Archaea has long been considered as strong argument in favour of the archaeal nature of the eukaryotic ancestor; new data restricts this argument to polyploid Archaea.

Importantly, our hypothetical scenario, in which the acquisition of mitosis by amitotic polyploid proto-eukaryotes was the key event in the evolution of eukaryotic sex, immediately suggests a historical explanation for several key features of genetic architecture and early evolution of eukaryotes, including the presence of multiple chromosomes, high level of genetic redundancy, and mass acquisition of new families of paralogous genes by the basal eukaryotes [16, 47].

The proposed extensive neo- and sub-functionalization of the redundant gene copies after the acquisition of mitosis implies that proto-eukaryotes at this stage of their evolution must have experienced considerable expansion of their functional genome size. This means that their per-genome deleterious mutation rate must have increased (given that per-nucleotide mutation rate remained constant). Thus the development of novel adaptations aimed to retain frequent between-individual recombination (which is an efficient remedy against genetic degradation) would still be favoured by selection.

In this paper, we discuss only the origin of eukaryotic sex and mitosis and not of the other essential eukaryotic traits such as mitochondria, nuclear envelope or endoplasmic reticulum. Our model was not aimed to provide arguments in favour of (or against) either of the numerous hypothetical scenarios of eukaryogenesis present in the literature. However, it is tempting to suggest that the acquisition of mitosis and eukaryotic sex by a polyploid archaeal ancestor, which provided extraordinarily high level of genetic redundancy coupled with an efficient mode of frequent and accurate between-individual recombination, was among the first and the most fundamental events that paved the way to many other evolutionary novelties.

There are several limitations to our computer simulation that should be aknowledged. Polyploidy can improve the efficiency of DNA repair by homologous recombination [21], resulting in lower mutation rate in polyploids compared to monoploids. Our model does not account for this effect, which can, at least theoretically, make the long-term disadvantages of polyploidy less prominent. However, there seems to be no strict correlation between polyploidy and resistance to double strand DNA breaks [11]; moreover, DNA break repair by homologous recombination may be risky in polyploid organisms because each DNA end will have multiple partners [22]. Another apparent limitation is that we consider only those beneficial mutations which are dominant, and only those deleterious mutations which are recessive (which is probably true for the majority of loss-of-function mutations). Taking into account dominant deleterious and recessive beneficial mutations would lessen the short term advantage of polyploidy; moreover, in this case, the evolutionary effects of polyploidy would be similar to those of having larger genome and higher ***U***_***genome***_, as discussed in the Methods section.

## Conclusion

We argue that eukaryotic sex evolved in polyploid amitotic Archaeal ancestors as a result of gradual evolution of adaptations aimed to minimize the genetic costs of polyploidy, which probably became especially high in mutagenic shallow-water environments of the Early Proterozoic [4]. Different adaptations of this kind may have evolved sequentially in a single lineage, or maybe, more probably, in different lineages, and then were combined via LGT.

We suggest the following scenario for the emergence of eukaryotic sex.

1. Like many extant Euryarchaeota, archaeal ancestors of eukaryotes (proto-eukaryotes) were polyploid and amitotic, i.e., they possessed no mechanism of accurate and precise chromosome segregation, although they were probably capable of relatively precise genome copy number control. Proto-eukaryotes were not necessarily related to Euryarchaeota. New genomic data suggest that eukaryotes branch within another, newly discovered archaeal lineage, Lokiarchaeota, whose ploidy is not known [48, 49]. However, it is possible that Lokiarchaeota are polyploid, because polyploidy in Archaea is correlated with the presence of histones [19], and the latter are present in Lokiarchaeota [50]. Adaptive significance of polyploidy could be manifold, including efficient repairing of multiple double strand DNA breaks by homologous recombination.
2. In highly mutagenic environment, polyploidy increases the risk of genetic degradation and extinction, because it decreases the efficiency of purifying selection against recessive deleterious alleles. In amitotic polyploids, this results in high segregation load. Therefore there could be a selection pressure to develop adaptations aimed to diminish the genetic costs of polyploidy.
3. Proto-eukaryotes probably evolved several different adaptations aimed to lessen the evolutionary costs of polyploidy, including ploidy cycles, equalization of genome copies by gene conversion, and extensive LGT between related (conspecific) cells. The latter occurred via cytoplasmic bridges formed in the course of mating (as described in *H. volcanii*) or maybe via temporary cell fusion, which *H. volcanii* is probably also capable of [10].
4. One of the most efficient modes of LGT, potentially available to polyploid Archaea, is the exchange of complete individual chromosomes between cells, coupled with homologous recombination between different chromosomes within cells. Proto-eukaryotes probably used this strategy extensively. In order to make frequent chromosomal exchange evolutionary stable, homologous recombination must be symmetrical (crossing over) rather than asymmetrical (gene conversion), because in the latter case ‘selfish’ modifier genes that prohibit recombination spread easily in the gene pool [8]. Therefore we suggest that proto-eukaryotes developed frequent (and probably reciprocal) exchange of individual chromosomes coupled with crossing over. In the course of evolutionary optimization of this process, circular chromosomes must have transformed into linear ones, because circular chromosomes are not fit for crossing over (they cannot separate properly if the number of crossovers is odd).
5. Later some proto-eukaryotes developed mitosis (i.e., a mechanism of accurate and precise chromosome segregation which ensures that each daughter cell receives exactly one copy of each parental chromosome). Among all possible adaptations for lessening the genetic costs of polyploidy, mitosis is the most radical one, because it completely removes the risk of segregation load accumulation imposed by polyploidy.
6. Initially, mitotic proto-eukaryotes retained all the benefits of frequent and more or less random chromosomal exchange, but later a new problem arose. Mitosis made multiple genome copies of proto-eukaryotes free to diversify, specialize, acquire new functions and accumulate differences from each other. This tendency came into conflict with the continued practice of random chromosome exchange and shuffling. Thus there was a selection pressure to (1) restrict recombination only to chromosome pairs with a high level of homology, (2) to prevent the exchange of different (mutually irreplaceable) chromosomes, and (3) to ensure that all chromosomes are involved in recombination with reasonable frequency. This eventually resulted in the development of tightly regulated cell fusions, homologous chromosome pairing (homolog synapsis) and meiosis [3]. Another inevitable result of this evolutionary transition was the development of more efficient mate recognition systems and the emergence of ‘biological species’.

One testable and very specific prediction that follows from our hypothesis is that there must have been mass acquisition of new gene families (sets of paralogous genes) near the base of eukaryotic lineage, because the acquisition of mitosis by an amitotic polyploid microbe immediately removes segregational constraints that prohibit the independent evolution (including sub-functionalization and neo-functionalization) of the gene copies located on different chromosomes. Strikingly, comparative genomics appears to confirm this prediction [47]. The origin of eukaryotes from polyploid prokaryotes that acquired mitosis thus may account, at least partially, for the puzzling fact that *“the characteristic eukaryotic complexity arose almost ‘ready made’, without any intermediate grades seen between the prokaryotic and eukaryotic levels of organization”* [16].

## Reviewers’ comments

### Reviewer’s report 1: Eugene V. Koonin, The National Center for Biotechnology Information, National Library of Medicine, National Institutes of Health, Bethesda, Maryland, USA

Markov and Kaznacheev develop the hypothesis that eukaryotic cell division mechanisms evolved from the segregation mechanisms of archaeal polyploid genomes. The hypothesis is explored using a mathematical model that involves a cost-benefit analysis of polyploidy, recombination mechanisms and the emerging mitosis. As far as I can see, the model is thoughtfully constructed and correctly analyzed. The conclusions are quite interesting, and I particularly like the idea that multiple eukaryotic chromosomes evolved from the initially identical chromosomes of archaeal polyploid. The authors correctly note that this scenario is compatible with the burst of duplication at the onset of eukaryotes. To the best of my knowledge, this is a new idea, and I find it quite plausible and promising. So this paper is, in my opinion, a useful and potentially important contribution to the field of eukaryotic origins and evolution which is in a dire need of fresh ideas. My only criticism that could qualify as major is that the title of the paper does not seem to actually reflect the content. Mostly, the analysis pertains to the origin of mitosis not sex.

I suggest that the authors change the title to "Evolutionary consequences of polyploidy in prokaryotes and the origin of mitosis and meiosis" (or another phrase to that effect).

Author’s response: *We highly appreciate these comments. We have changed the title as suggested by the reviewer.*

The authors repeatedly write about "evidence in favour of the scenario" (end of the abstract), "evidence that confirm" (end of the Discussion) and other phrases to that effect. This is not good practice. Evidence can only be compatible with a hypothesis/scenario not "confirm" it. The phrase "recent archaea" is strange - do the authors mean "extant archaea"?

Author’s response: *We have changed the wording as suggested by the reviewer. Paleontologists often use ‘recent’ and ‘extant’ as synonyms, e.g. “fossil and recent Cephalopoda”.*

### Reviewer’s report 2: Uri Gophna, Tel Aviv University, Israel

This is a highly original and thought provoking simulation study that will pave the way to future investigations about the role of polyploidy, and the various ways for resolving its costs, in eukaryogenesis.

To the best of my knowledge there has been no demonstration of conjugation in Hfx, but rather a process of gene exchange through broad cytoplasmic bridges more compatible with cell fusion was shown by Rosenshine et al. 1989. The bridges shown in that work as well as large-scale recombination events reported in Naor et al., 2012 are not compatible with a process similar to bacterial conjugation (i.e. single strand transfer trhough a narrow channel from donor to recipient). The authors can and should use the same terminology for it in the abstract and introduction as they do in the discussion (i.e. exchange through bridges, potentially cell fusion).

Author’s response: *We agree that using the term ‘conjugation’ to describe the mating system of Haloferax can be misleading. Although the term is commonly applied to different types of mating in different organisms (e.g., ciliates), Rosenshine et al. 1989* [9] *and other authors use the terms “mating”, “genetic transfer system”, or “genetic exchange system”, rather than “conjugation”. We have changed the wording as suggested by the reviewer*.

Two important points should be at least briefly discussed 1. Because poly-ploidy can improve accuracy of DNA repair (via accurate HR), polyploid may have lower mutation rate than monoploids. 2. A ploidy cycle can occur naturally in organisms that respond to phosphate availability by changing ploidy levels, such as Hfx volcanii (see Zerulla et al., which the authors cite).

Author’s response: *We are thankful for these comments. We have included these considerations in the revised version of the manuscript. Interestingly, Delmas et al.* [22] *argue that double strand break repair by HR is potentially hazardous to polyploid organisms, and demonstrate that Mre11-Rad50 prevents the repair of DSB by HR in Haloferax, “allowing microhomology-mediated end-joining to act as the primary repair pathway”.*

"The conjugation of the halophilic archaeon Haloferax volcanii probably represents an intermediate stage between typical prokaryotic sex and amphimixis" - see my objection to the term conjugation above, and also a term such as "amphimixis" should be avoided in an abstract.

Author’s response: *We have removed the term ‘conjugation’ from the revised version of the manuscript. We have also rephrased the abstract so as to avoid the term ‘amphimixis’, which is introduced later on in the text.*

Introduction "This obstacle can be partially overcome by ongoing strong selection against homozygotes [12, 31]" should be "In the lab, this obstacle can be partially overcome by ongoing strong selection against homozygotes [12, 31]", since no such natural selection has been observed.

Methods The assumption that all beneficial/best alleles are dominant over the less fit allele is a limitation of the model that should be acknowledged: in many cases this will not be the case and one bad allele may "poison" a complete complex (dominant negative/dominant lethal phenotype)

‘gene concersion’ - should be ‘gene conversion’

Discussion "because larger genome (G)' should be "because a larger genome (G)"

Author’s response: *We have made appropriate corrections. We have included the discussion of limitations of the model in the Discussion section.*

### Reviewer’s report 3: Armen Mulkidjanian, University of Osnabrück, Germany

The paper of Markov and Kaznacheeev describes a model for numerical simulations of reproduction of polyploid cells. The modeling data indicate that polyploidy per se might lead to adverse effects, and that such effects can be counteracted by different means, including an accurate chromosome distribution during cell division (mitosis). Based on the modeling, the authors hypothesize that mitosis may have emerged in a polyploid archaeal cell under strong selective pressure from oxygenated environment. The model is illustrative and might be useful, particularly, in teaching.

Several points deserve further consideration: 1) The model demonstrates the advantage of mitosis; as far as I could understand, the meiosis was not modeled. Then, however, the subject of the paper is the emergence of mitosis and not the emergence of sex. Sex is a more complex phenomenon that mitosis; the sex, ultimately, includes also sexual differentiation, which was not discussed in the manuscript at all. Therefore, the subject of the paper should be defined in a more precise way, both in the abstract and in the title. Definitely, the paper is not about the phenomenon of sex as a whole, but about tentative first steps towards the sexual reproduction.

Author’s response: *Although the meiosis was not modeled, we argue that the emergence of mitosis in a polyploid prokaryote results in chromosome specialization which, in turn, can result in selection pressure to develop accurate homolog pairing* [3]*. We agree that, although our initial aim was to elucidate the origin of sexual reproduction (amphimixis), our results pertain mostly to the origin mitosis and, partially, meiosis (homolog synapsis). Thus we have changed the title (as suggested by the first reviewer as well) and rephrased the abstract.*

2) The presented model shows a clear advantage of mitosis (at least, for a particular set of parameters) as compared to polyploidy. Then, however, it remains unclear why all polyploid archaea did not switch to the elaborated mitosis but continued to distribute their chromosomes between the two dividing cells in a random, unsophisticated way. One possibility is that there were some other factors, which favored the emergence of mitosis in a specific lineage and, apparently, were absent in other polyploid Archaea. These factors deserve discussion.

Author’s response: *We can only speculate that these ‘other factors’ probably included highly mutagenic environments of the Early Proterozoic shallow-water microbial communities, when O*_*2*_*levels started to rise, but there was still no ozon layer to shield these habitats from UV radiation, resulting in high ROS concentration* [4]*. Other possible factors are the invasion of type II self-splicing introns from the mitochondrial ancestor* [15]*, and the supposedly rapid increase of gene complement and functional genome size during the eukaryogenesis* [8, 16]*. All these factors could contribute to the increased per-genome mutation rate (U*_*genome*_*) and thus favor the emergence of mitosis in proto-eukaryotes. Other polyploid archaeal lineages probably did not experience this combination of factors and thus had lower U*_*genome*_*. Therefore, other strategies (e.g., extensive LGT and equalization of genome copies by gene conversion) were sufficient to remove the risks of polyploidy. Moreover, the molecular machinery of mitosis must be costly. Its emergence requires the presence of components of cytoskeleton which were probably present in proto-eukaryotes and their closest relatives* [48]*, but not in the other polyploid archaea.*

3) As authors have noted, polyploid species are found within Euryarchaeota. Eukaryotes, however, show more similarity to Crenarchaeota, which are typically monoploid or, sometimes, diploid. Last year, at least two groups of Archaea, namely Lokiarchaeota and Thorarchaeota, were identified by means of metagenomics as tentative transition forms between Crenarchaeota and eukaryotes. Since these organisms are not available in pure cultures yet, it is unclear whether they are polyploid or not. However, the closeness to Crenarchaeota makes polyploidy of Lokiarchaeota and Thorarchaeota unlikely. Some comments on the obvious conundrum between the suggested scenario for the emergence of eukaryotic meiosis from polyploidy of Archaea and the apparent monoploidy of Crenarchaeota would be appreciated.

Author’s response: *This is an important issue. Indeed, if eukaryotes evolved from polyploid Archaea, we should expect that the closest extant archaeal relatives of eukaryotes, such as Lokiarchaeota* [48]*, are polyploid. As noted by the reviewer, there is as yet no direct information on ploidy in Lokiarchaeota. However, there is circumstantial evidence. Spaans et al. have recently demonstrated a rather strict correlation between the presence of histones and polyploidy in Archaea* [19]*. Therefore, if Lokiarchaeota are polyploid, they are expected to have histones. We were delighted to find out that this was the case. In a paper published in December 2015, Henneman and Dame report the identification of histones and other chromatin proteins in the genomes of Lokiarchaeota; interestingly, some residues in these histones are shared between Lokiarchaeota and eukaryotes, but not with Euryarchaeota, while others are shared with histones from Euryarchaeota but not with eukaryotes* [50]*. The presence of histones in Lokiarchaeota is compatible with the hypothesis that Lokiarchaeota are polyploid. A putative histone is also found in the genome of Candidatus Thorarchaeota archaeon SMTZ1-45 (**http://www.ncbi.nlm.nih.gov/protein/KXH71038.1**).*

*The authors would like to thank the reviewers for their constructive comments.*

## Availability of materials

Project name: Microbe

Project home page: https://github.com/alamar/microbe

Archived version: 10.5281/zenodo.46663

Operating systems: Platform independent

Programming language: Java

Other requirements: Java 7 or higher for running models; Maven as build tool

License: Open Source under Apache Software License

This is the implementation of the computer model devised for this paper. It is a command-line Java program that simulates population specified by a set of parameters provided in Java Properties file, editable as text. It produces a text file, tab separated, with simulation results, and a mean fitness chart. Most figures for this paper were generated programmatically by our computer model.

Please use “Download ZIP” link on the project homepage to download the program, complete with README file explaining how to use it, source code, batch files for running model under Windows, Mac OS X and Linux, and bundled model parameters for all simulations mentioned in this paper.

## Abbreviations

DSB, double strand break; HR, homologous recombination; LGT, lateral gene transfer; ROS, reactive oxygen species
